# Genomic signatures of radiation stress adaptations in *Kocuria rhizophila*: insights from strain 301 of the Jáchymov radon springs

**DOI:** 10.3389/fmicb.2026.1814458

**Published:** 2026-07-08

**Authors:** Elizaveta Timkina, Andrea Palyzová, Helena Marešová, Sofía G. Zavala-Meneses, Olga Mat́átková, Irena Jarošová Kolouchová

**Affiliations:** 1 Department of Biotechnology, University of Chemistry and Technology, Prague, Czechia; 2 Institute of Microbiology, Czech Academy of Sciences, Prague, Czechia

**Keywords:** antioxidant defense, genome assembly, irradiation resistance, *Kocuria*, stress adaptation

## Abstract

Several strains of *Kocuria rhizophila* have been reported to tolerate ionizing radiation and other environmental stresses; however, this phenotype is unevenly distributed across the species. Here, we characterize *K. rhizophila* strain 301, isolated from chronically radioactive radon springs in Jáchymov (Czech Republic). Strain 301 displayed exceptional stress resilience, retaining approximately 10% viability after exposure to 1.0 kGy of γ-irradiation, and maintaining ~20% survival following 30 days of desiccation. These responses sharply contrasted with the pronounced sensitivity of the type strain *K. rhizophila* TA68. Complete genome sequencing produced a single circular chromosome of 2.77 Mbp. Comparative genomic analyses revealed extensive duplication of genes involved in DNA repair, antioxidant defense, and metal ion homeostasis, including multiple paralogs of *uvrA, uvrD, sodA*, and Mn/Fe transport systems. In contrast to the paradigm established by *Deinococcus radiodurans*, strain 301 lacks radiation-specific or lineage-exclusive genes, instead suggesting that resilience may be associated with quantitative reinforcement of conserved cellular pathways. Pan-genome analysis further demonstrated a closed *K. rhizophila* pangenome, with strain 301 forming a distinct phylogenetic lineage.

Together, these findings position *K. rhizophila* 301 as a model system of adaptation to chronic radiation exposure and illustrate how sustained environmental pressure may promote modifications and adaptations based on core functions rather than novel innovative genetic traits.

## 1 Introduction

Ionizing-radiation-resistant bacteria (IRRB) are defined as non-spore-forming microorganisms that combine the ability to protect cytosolic proteins from oxidative damage with the capacity to survive extensive DNA double-strand breaks (DSBs). Functionally, this group includes strains capable of withstanding acute ionizing radiation doses exceeding 1.0 kilogray (kGy), corresponding to *D*_10_ values above this threshold (Sghaier et al., 2008). Most current understanding of microbial radiation resistance originates from studies of *Deinococcus* species, particularly *D. radiodurans*, which has served as the canonical model for elucidating mechanisms of DNA repair, oxidative stress management, and protein protection (Timmins and Moe, 2016; Daly, 2009; Krisko and Radman, 2013; Rajpurohit et al., 2021; Sharma et al., 2022b, 2024) However, radioresistance is not restricted to *Deinococcus*. Increasing evidence demonstrates that representatives of the phylum Actinomycetota also exhibit pronounced tolerance to ionizing radiation (Bagwell et al., 2008; Egas et al., 2014; Suzuki et al., 1988; Phillips et al., 2002).

Comparative genomic studies of IRRB have revealed that radiation resistance is typically not conferred by novel DNA repair pathways but rather by evolutionary refinement and reinforcement of conserved cellular mechanisms. Essential DNA repair systems, including recombination- and excision-based pathways, frequently show signatures of positive selection, indicating adaptive fine-tuning driven by chronic exposure to DNA damage (Bruckbauer et al., 2020; Sghaier et al., 2008). This strategy is exemplified by *Kineococcus radiotolerans*, whose genome encodes duplicated DNA repair genes alongside oxidative stress efences and metal ion homeostasis systems, collectively supporting survival in nuclear waste environments (Bagwell et al., 2008). Natural radioactive springs, where microorganisms experience sustained exposure to ionizing radiation combined with geochemical stressors, therefore represent valuable systems for studying radiation adaptation under ecologically relevant conditions.

The radon springs of Jáchymov (Czech Republic), formed by groundwater percolation through uranium-rich ore deposits, rank among the most radioactive natural aquatic environments worldwide. These springs are characterized by exceptionally high radon activity (5–23 kBq·L^−1^) and mildly thermal water temperatures (27 °C−37 °C) (Weidler et al., 2007; Asker et al., 2007; Asgarani et al., 2012; Brugger et al., 2005; Krejbichova, 1999; Golias et al., 2022). These values correspond to elevated radiation exposure conditions in radon-rich environments, where indoor radon concentrations in spa settings of Jáchymov radon springs are associated with effective radiation doses typically reported in the range of mSv per year, depending on exposure conditions.

Naturally radioactive springs occur worldwide and exhibit a wide range of radon activities and physicochemical properties. For example, the Franz-Josef-Quelle in Bad Gastein (Austria) is a thermal spring (45.6 °C, pH 8, mineralization ~350 mg·L^−1^) with a radon activity of approximately 0.3 kBq·L^−1^ (Weidler et al., 2007). In contrast, highly radioactive thermal springs such as Paralana (South Australia) reach temperatures up to 63 °C and display substantially higher radon activity, ranging from 2 to 5.8 kBq·L^−1^ in water (Anitori et al., 2002). The values observed in Jáchymov springs thus fall within the upper range of naturally occurring radon-rich environments and although radioactive springs occur globally, Jáchymov is distinguished by the unique combination of extreme radon concentrations and uranium-rich geology, creating long-term selective pressure on resident microbial communities.

Members of the genus *Kocuria* (Actinomycetota) are repeatedly isolated from radiation-rich environments, suggesting a strong ecological association with habitats dominated by physical and chemical stressors. *Kocuria* strains have been reported from radioactive springs in Ramsar and Misasa (Asker et al., 2007; Gholami et al., 2015), as well as from anthropogenic radioactive environments such as nuclear reactor cooling pools, where *Kocuria* was among the few genera capable of persistent survival (Petit et al., 2023). In addition, several desert-derived *Kocuria* species (*K. rosea, K. indica, K. tufuensis, K. salina*) display marked tolerance to UV-C irradiation (Liu et al., 2022). Collectively, these observations support the view that *Kocuria* spp. Are broadly pre-adapted to environments characterized by ionizing radiation, ultraviolet exposure, oxidative stress, and chemical challenge (Rezanka et al., 2025).

Despite the ecological breadth of the genus, enhanced radiation resistance has been described in only a limited number of *Kocuria* strains (Liu et al., 2022; Guesmi et al., 2021; Rasuk et al., 2017; Gholami et al., 2015; Mehrabadi et al., 2016; Shukla et al., 2007; Timkina et al., 2024). A recent polyphasic analysis of *Kocuria rhizophila* PT10, isolated from irradiated roots of *Panicum turgidum* in the Tunisian Sahara, provided the first in-depth characterization of radiation tolerance in this species (Guesmi et al., 2021). Genome sequence of strain 301 revealed a complete complement of conserved DNA repair and oxidative stress response genes, together with multiple determinants implicated in protein protection under radiation-induced oxidative stress, including antioxidants, pigments, and Mn/Fe transport systems. Such protein-centered protection mechanisms are increasingly recognized as central to radiation resistance in extremophiles (Fredrickson et al., 2008). By integrating ecological context with phenotypic characterization and comparative genomics, *K. rhizophila* 301 provides a valuable model for studying adaptive strategies that enable microbial persistence in chronically radioactive environments.

## 2 Materials and methods

### 2.1 Bacterial strains and culture conditions

Isolate 301 was obtained from spring C1 in Jáchymov (50.3726072 N, 12.9116558 E), an environment characterized by elevated levels of natural radioactivity (Timkina et al., 2022). Strain 301 was grown in half-strength TSB and TSA media, that were prepared by diluting standard formulations (Sigma-Aldrich, St. Louis, MO, USA, cat. Nr. 22092) to 50% concentration. Diluted medium was used to maintain consistency with the original isolation conditions of strain 301, which was recovered from a nutrient-poor environment using diluted TSB. Type strain of *K. rhizophila* TA68 (=CCM 4950 = DSM 11926), grown in TSB broth and TSA agar, was used as reference strain for biochemistry and resistance tests *in vivo*. *D. radiodurans* R1 (=CCM 1700T = DSM 20539), grown in B8 broth (yeast extract (5 g/L), peptone (5 g/L), and glucose (10 g/L), pH 7.2), and *Escherichia coli* CCM 4517 (=DSM 1116), grown in TSB broth, were used for resistance tests as positive and negative control strains, respectively. All *K. rhizophila* strains and *D. radiodurans* R1 were incubated at 30 °C under aerobic conditions for 72 h. *E. coli* CCM 4517 was grown at 37 °C for 24 h. At the end of cultivation (all strains we cultivated into late exponential phase) the cells were harvested by centrifugation (10 min, 9,000 RCF), washed twice with sterile physiological saline solution and bacterial suspensions of optical density (measured at 600 nm) 0.5 were prepared (equal to 10^7^-10^8^ of viable cells) for further tests.

### 2.2 Resistance to gamma irradiation

Bacterial suspensions of all studied strains in microtubes (1 ml) were irradiated using a circular electron accelerator microtron MT25. The device generates bremsstrahlung gamma radiation, which is produced by the deceleration of an electron beam as it passes through a tungsten target located behind the device's exit window. The resulting radiation has a continuous energy spectrum with a maximum energy of 16.5 MeV. Five doses of gamma radiation were administered: 0.25, 0.5, 1.0, 1.5, and 2.0 kGy. The absorbed dose was determined using a calibrated ionization chamber (TN34045, PTW Freiburg, Germany) and a Keithley 617 electrometer (Keithley, USA, ensuring consistent dosimetry of the irradiation conditions against all studied samples). A non-irradiated bacterial suspension served as control. Viable bacterial cell counts were determined by serially tenfold diluting samples and spreading them on agar plates, followed by incubation at either 30 °C or 37 °C for 48–72 h. *D*_10_ values were estimated from linear regression of log-transformed survival data and defined as the irradiation dose required to reduce viability by one order of magnitude.

Fitted regression curves were as follows: *D radiodurans*: *y* = 1.435×, *R*^2^ = 0.99; *E. coli*: *y* = 3.0982×, *R*^2^ = 0.98, *D*_10_ = 0.323 kGy; *K. rhizophila* 301: *y* = 1.0246×, *R*^2^ = 0.99, *D*_10_ = 0.976 kGy; *K. rhizophila* TA68: *y* = 2.3215×, *R*^2^ = 0.97, *D*_10_ = 0.431 kGy.

Experiments were conducted in triplicates and repeated independently three times.

### 2.3 Biochemical characterization

Bacterial suspension (100 μl) was pipetted into each well of a Biolog GEN III MicroPlate containing various carbon sources and stress factors (osmotic stress, antibiotics, detergents and permeability stress, heavy metals, or toxic compounds). The plate, covered with a plastic lid and wrapped in parafilm, was incubated at 30 °C for 2 days. The chromogenic reagent MTT was dissolved in a sterile PBS solution to a concentration of 2 mg/ml. A 25 μl of this MTT solution was pipetted into each well. The plate was incubated in the dark for 2 h. After incubation, the contents of the plate were thoroughly pipetted into a new microtiter plate with larger well volumes. To prepare the MTT washing solution, acetic acid (2% solution in PBS) was mixed with DMF (dimethylformamide) in a volume ratio of 6:4. Then, SDS (sodium dodecyl analyzed) was added to a final concentration of 160 g/L, and the pH was adjusted to 4.7. A 100 μl of the MTT washing solution was pipetted into each well of the new microtiter plate, and the well contents were gently mixed with a pipette. The washing solution dissolved the blue formazan crystals formed from MTT due to the metabolic activity of the microorganism. Subsequently, the absorbance of the plate was measured in a Tecan microplate reader at 570 nm. The measured data were nalysed using MS Excel.

### 2.4 Genome sequencing and assembly

Genomic DNA of strain 301 was extracted from bacterial cells using the ZymoBIOMICS™ DNA Miniprep Kit (Zymo Research, Irvine, CA, USA) according to the manufacturer's instructions. For genome extraction, cells were cultivated in LB medium for 48 h at 30 °C to obtain sufficient biomass and high-quality genomic DNA suitable for sequencing. Whole-genome sequencing was performed by an external sequencing facility (SEQme, Dobríš, Czech Republic) using Oxford Nanopore MinION FC, single-end (DS-210) and Illumina Paired-end, 150b (DS-150S) platforms. The Oxford Nanopore library was prepared using the Ligation Sequencing Kit SQK-LSK109, yielding a total of 77,375 reads with a mean read length of 13,440 bp. The Illumina paired-end library (2 × 150 bp) was prepared using the NEBNext Ultra II DNA Library Prep Kit (New England Biolabs, Ipswich, MA, USA) for Illumina, generating 15,731,742 reads.

Raw Oxford Nanopore data were basecalled using program Guppy v3.2.10 in high-accuracy mode. Sequencing run and quality of reads were checked using NanoPlot v1.30.1 (De Coster and Rademakers, 2023). Filtering according to the quality and read length was performed by NanoFilt v2.7.1 (De Coster et al., 2018). Reads shorter than 1000 bp and/or with lower quality than Q10 were filtered out from the dataset. Quality of Illumina reads was evaluated using programs FastQCv0.11.8 and MultiQC v1.9 (Ewels et al., 2016). Adapter sequences and reads with lower quality than Q30 were removed using Trim Galore v0.6.6. Genome assembly was performed by program Canu v1.4 (Koren et al., 2017), a long-read assembler optimized for Oxford Nanopore sequencing data, particularly suited for handling noisy reads. The resulting assembly was subsequently polished using Illumina data to improve accuracy. The correction of nanopore reads was done in the first step. Secondly, nanopore data were assembled and trimmed in the next step of the Canu pipeline. Trimmed data from Illumina sequencing were mapped to the relevant draft assembly using BWA aligner v0.7.17-r1188. Samtools toolkit v0.1.20 was used for manipulating mapped data (Li and Durbin, 2010). Assembly was polished by program Pilon v1.23 using output of mapped Illumina reads and draft of genome (Walker et al., 2014). This polishing was done in three rounds, producing the final assembly KOC301_pilon_round3. QUAST analysis of KOC301_pilon_round3 reported a single contig of 2,773,901 bp with a GC content of 70.59%, N50 equal to the assembly length, duplication ratio 1.019, and no misassemblies.

### 2.5 Taxonomic identification

The genome sequence of strain 301 was analyzed using the type (strain) genome server (TYGS, https://tygs.dsmz.de) for whole-genome based taxonomic analysis. Initial comparisons were performed against all available type strain genomes in the TYGS database using the MASH algorithm, resulting in the identification of the ten type strains with the smallest MASH distances (Ondov et al., 2016). In parallel, closely related taxa were identified based on 16S rRNA gene sequence similarity. The 16S rRNA gene sequence was extracted from the genome of strain 301 using RNAmmer and compared with corresponding sequences of all type strains available in the TYGS database using BLAST, allowing the selection of the top 50 matching type strains based on bitscore values (Lagesen et al., 2007; Camacho et al., 2009). Accurate intergenomic distances were subsequently calculated using the genome BLAST distance phylogeny (GBDP) approach employing the “coverage” algorithm and distance formula d5. These distances were used to determine the ten closest related type strain genomes, and digital DNA–DNA hybridization (dDDH) values between strain 301 and the selected type strains were calculated using the genome-to-genome distance calculator (GGDC) (Meier-Kolthoff et al., 2013).

### 2.6 Genome set selection

Following identification of the closest type strain, the NCBI Genome database was searched for publicly available genomes affiliated with this taxon. Only isolate genomes (excluding metagenome-assembled genomes) meeting quality criteria of ≥95% completeness and ≤ 5% contamination were considered. To capture ecological diversity, genomes derived from different habitats were preferentially selected, while redundant genomes from similar sources were excluded. Average nucleotide identity based on BLAST (ANIb) values were calculated *in silico* using the JSpeciesWS web server, and 29 genomes with the highest ANIb values were retained for subsequent comparative genomic analyses (Richter et al., 2016).

### 2.7 Genome annotation

Coding sequences (CDSs) were predicted by GeneMarkS-2 software (Lomsadze et al., 2018; Besemer and Borodovsky, 2005). Microscope platform was used for the genome annotation and comparative genomics (Vallenet et al., 2020). CGView tool was used for genome visualizing (Stothard et al., 2019). EggNOG-mapper provided the number of CDS classified by eggnog in at least one COG functional category (Cantalapiedra et al., 2021). Putative horizontally transferred genes [which are gathered in genomic regions (Region of Genomic Plasticity)] were identified based on a combination of following criteria:HGT features (tRNA hotspot, mobility genes), compositional bias, SIGI-HMM, and GC deviation computation in the query genome (Vernikos and Parkhill, 2006; Waack et al., 2006).

### 2.8 Analysis of genes related to DNA repair and oxidative stress

Genes associated with DNA repair (homologous recombination (HR), non-homologous end joining (NHEJ), mismatch repair (MMR), base excision repair (BER), nucleotide excision repair (NER), SOS response) and oxidative stress detoxification (catalase, superoxide dismutase, peroxidase, thioredoxin system, Mn-transporters) were identified based on published literature. Reference genomes of *E. coli* K12, *D. radiodurans* R1, *Mycobacterium tuberculosis* H37Rv, and *Kineococcus radiotolerans* SRS30216 served as benchmarks (Bagwell et al., 2008). For isolate 301, BLAST searches against the predicted proteome were performed, and genes were considered present when homologs were detected with *e*-values ≤ 1*e*^−6^ (Camacho et al., 2009; Choudhuri, 2014).

### 2.9 Pan-genome analysis

For the pan-genomic study, Anvio-8, a widely used software suite for analyzing and visualizing metagenomic data, was employed (Eren et al., 2021). Anvio-8 integrates various tools and workflows for comparative genomics, metagenomics, and pangenomics analyses, providing robust capabilities for exploring genomic diversity and evolutionary relationships within microbial communities. Initially, all selected genomes were formatted into an appropriate genome storage database. Subsequently, open reading frames were predicted using Prodigal (Hyatt et al., 2010). COG annotation and hidden Markov model (HMM) search were conducted. The pan-genome was constructed utilizing the Anvio pangenomics workflow. Phylogenetic tree construction was performed based on single-copy core genes (SCGs) within the selected genome set, employing the SCGs Bayesian Tree method. TrimAL was utilized by Anvio to eliminate nucleotide positions containing gap characters in more than 50% of the sequences. Finally, IQ-TREE software, employing the “WAG” general matrix model, was used to infer a maximum likelihood tree (Minh et al., 2020; Capella-Gutiérrez et al., 2009).

To quantitatively estimate pan-genome openness, a power-law regression model was applied to the growth of the pan-genome (Tettelin et al., 2008). The input was a matrix of gene families generated by Anvi'o. Random permutations of genome addition order (100 iterations) were simulated to compute the average pan-genome growth curve. The curve was fitted to the model $n_{pan}=\chi \times N^{\gamma }$, where the exponent γ indicates the degree of pan-genome openness. In parallel, the model Δ*n* = χ′ × *N*^−α^ was applied to estimate the decline in novel gene families, with α > 1 indicating a closed pan-genome. All calculations were performed in Python using the NumPy, SciPy, and Matplotlib libraries.

## 3 Results and discussion

### 3.1 Genomic characterization of isolate 301

**Figure 1:**
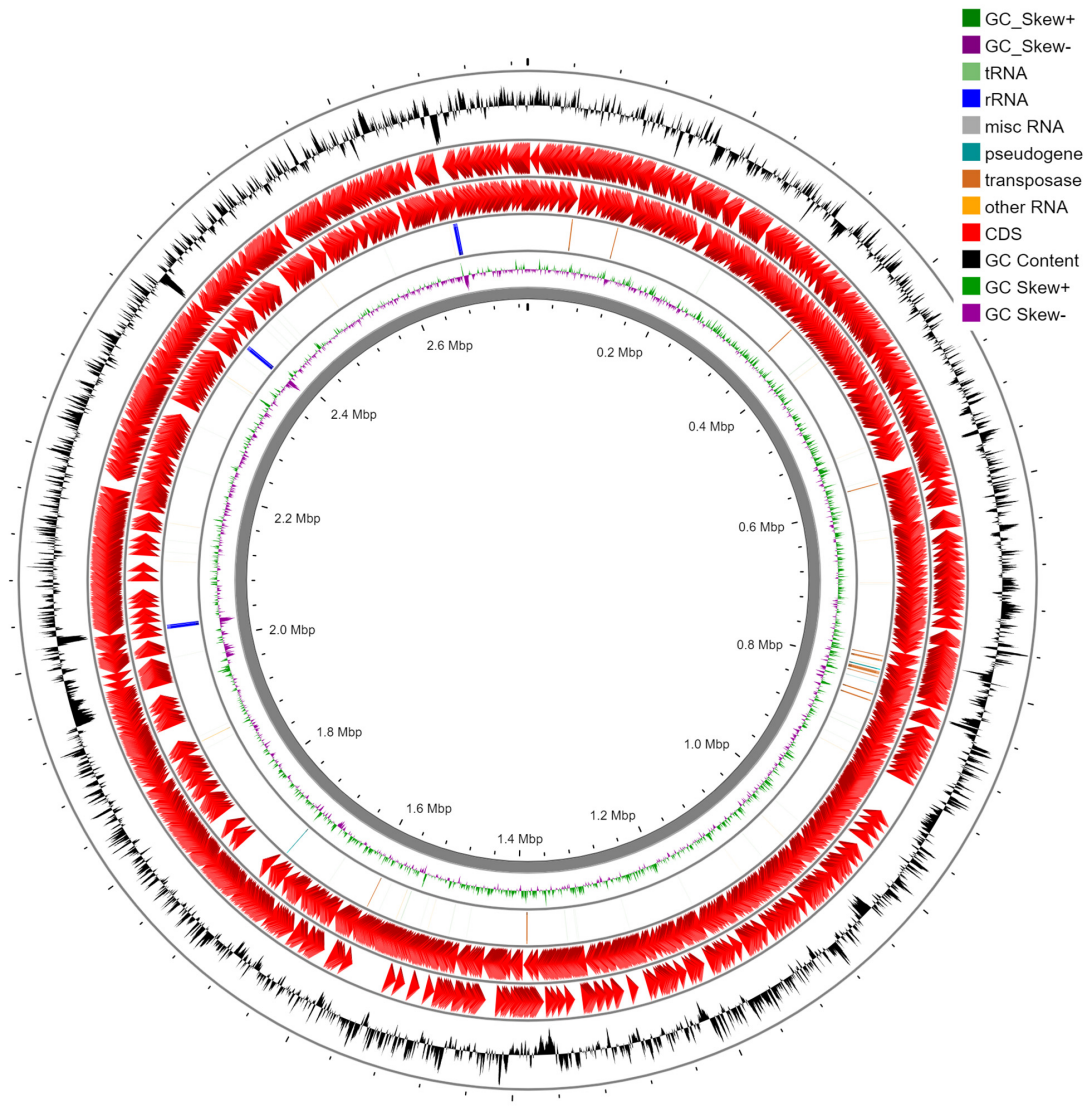
Graphical map of the *K. rhizophila* 301 chromosome. The circular map represents the complete genome of strain 301, showing the distribution of genomic features across the chromosome. From outer to inner rings, annotated coding sequences (CDSs) on the forward and reverse strands are indicated, followed by the positions of RNA genes (tRNA and rRNA). The inner rings display GC content and GC skew, highlighting local variations in nucleotide composition.

The genome of isolate 301 was sequenced using a hybrid approach combining long Oxford Nanopore reads with high-accuracy Illumina short reads. The hybrid assembly yielded a single contig of 2.77 Mbp, representing a continuous scaffold with no detected plasmids. Annotation predicted 2,497 coding sequences (CDSs), nine rRNA genes, and 46 tRNA genes, with a coding density of 89.03% and a GC content of 70.59% (Table 1). The circular genome map (Figure 1) illustrates the orientation of predicted CDSs, with rRNA, tRNA, and other RNA genes highlighted in blue, green, and orange, respectively.

**Table 1:** Proteogenomic features of isolate 301.

Genome size (bps)	2,773,901
G + C content (%)	70.59
Number of scaffolds	1
Number of contigs	1
CDS	2,497
Protein coding density (%)	89.03
rRNA genes	9
tRNA genes	46

**Figure 2:**
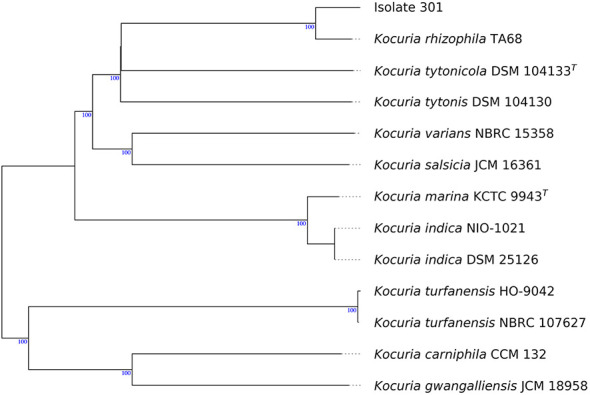
Phylogenetic tree based on whole-genome sequences showing the position of *K. rhizophila* isolate 301. The tree was reconstructed using genome BLAST distance phylogeny (GBDP) distances calculated from whole-genome sequences. Branch lengths are proportional to genomic distance as inferred by GBDP. The phylogenetic tree was generated using FastME v2.1.6.1 [33]. The analysis illustrates the evolutionary relationship of strain 301 to closely related taxa within the genus *Kocuria* and other selected reference strains.

To determine the precise taxonomic affiliation of isolate 301, phylogenomic analysis was performed using the TYGS platform. Whole genome sequence and the 16S rRNA gene extracted with RNAmmer (data not shown) were compared against available type strains, confirming the placement of isolate 301 within the species *K. rhizophila* (Figure 2). The closest relative was the type strain *K. rhizophila* TA68 (=CCM 4950 = DSM 11926), as shown in the phylogenetic tree reconstructed from the closest reference genomes. Whole-genome similarity metrics further supported this identification: digital DNA–DNA hybridization (dDDH) was 89.8% and OrthoANIu was 96.03% (Richter and Rosselló-Móra, 2009). Both metrics well above accepted thresholds for species-level assignment. These results unequivocally place isolate 301 within *K. rhizophila* and demonstrate its close genomic relatedness to the reference type strain.

Functional classification of CDSs into Clusters of Orthologous Groups (COGs) revealed representation across all major categories, with a substantial proportion of hypothetical proteins (Table 2). This likely reflects the limited genomic characterization of stress-adapted *Kocuria* strains and suggests the presence of unexplored genetic diversity potentially relevant to environmental adaptation. Importantly, the availability of a complete, closed genome provides a framework for assessing how quantitative changes within a conserved genetic repertoire may contribute to stress-associated phenotypes, rather than adaptation via gene acquisition.

**Table 2:** Number of genes associated with the general COG functional categories.

COG category	COG category description	CDS	%
D	Cell cycle control, cell division, and chromosome partitioning	37	1.44%
M	Cell wall/membrane/envelope biogenesis	111	4.33%
N	Cell motility	5	0.20%
O	Post-translational modification, protein turnover, and chaperones	82	3.20%
T	Signal transduction mechanisms	76	2.97%
U	Intracellular trafficking, secretion, and vesicular transport	35	1.37%
V	Defense mechanisms	36	1.41%
B	Chromatin structure and dynamics	1	0.04%
J	Translation, ribosomal structure, and biogenesis	171	6.68%
K	Transcription	190	7.42%
L	Replication, recombination and repair	157	6.13%
C	Energy production and conversion	137	5.35%
E	Amino acid transport and metabolism	243	9.49%
F	Nucleotide transport and metabolism	83	3.24%
G	Carbohydrate transport and metabolism	147	5.74%
H	Coenzyme transport and metabolism	103	4.02%
I	Lipid transport and metabolism	112	4.37%
P	Inorganic ion transport and metabolism	161	6.29%
Q	Secondary metabolites biosynthesis, transport, and catabolism	48	1.87%
S	Function unknown	626	24.44%

### 3.2 Phenotypic characterization of isolate 301

Biochemical profiling revealed that isolate 301 possessed a broader metabolic capacity than the type strain *K. rhizophila* TA68 (CCM 4950), utilizing additional substrates such as p-hydroxyphenylacetic acid, γ-aminobutyric acid, D-maltose, D-trehalose, D-glycerol, N-acetyl-D-glucosamine, L-histidine, L-pyroglutamic acid, L-malic acid, inosine, and D-serine (Kovacs et al., 1999). Both strains shared the ability to metabolize several common substrates and exhibited comparable resistance to multiple stressors, including antibiotics, salts (1%−4% NaCl), and pH 6. By contrast, TA68 showed greater tolerance to basic physical conditions, sustaining growth at pH 5 and 8% NaCl, conditions inhibitory for isolate 301. Enzymatic activities detected by API ZYM (bioMérieux, Marcy-l'Étoile, France) were identical in both strains (see Supplementary Table 1). These results indicate moderate metabolic plasticity but do not explain the pronounced radiation resistance of isolate 301, supporting the hypothesis that the observed stress tolerance may involve specific protective and repair-associated systems rather than general metabolic breadth alone.

### 3.3 Resistance to irradiation and environmental stress

**Figure 3:**
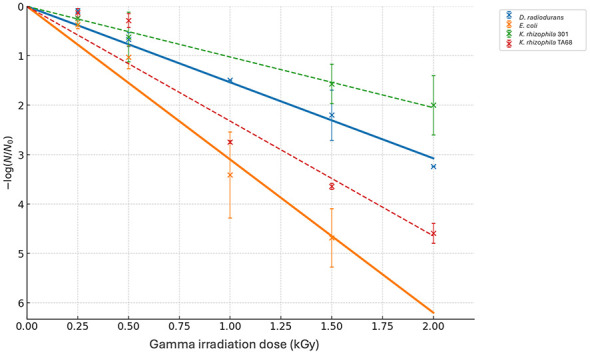
Gamma irradiation inactivation curves of isolate 301 and reference strains. Survival is expressed as log_10_(*N*/*N*_0_) as a function of absorbed dose (kGy), where N_0_ represents the initial viable cell count and N the viable count after irradiation. Lines represent linear regression fits used to estimate *D*_10_ values. Data represent mean ± standard deviation of three independent experiments, each performed in triplicate.

Evaluation of gamma resistance showed that *K. rhizophila* 301 retained ~10.2 ± 0.3% viability at 1.0 kGy, compared with only 0.18 ± 0.04% for the type strain *K. rhizophila* TA68 and 0.039 ± 0.006% for *E. coli* CCM 4517 (Figure 3). These results indicate that pronounced radioresistance is not uniformly distributed across the species and likely reflects strain-specific adaptation.

*K. rhizophila* 301 also exhibited strong irradiation tolerance (studied up to 2 kGy), comparable to *D. radiodurans* (under the experimental conditions used in this study) and markedly higher than *E. coli*, and retained ~20% viability after 30 days of desiccation (Timkina et al., 2022). Based on linear regression of –log(*N*/*N*_0_) survival curves, the estimated *D*_10_ values were 1.0 kGy for *K. rhizophila* 301, 0.7 kGy for *D. radiodurans*, 0.4 kGy for *K. rhizophila* TA68, and 0.3 kGy for *E. coli* under the experimental conditions used. Highly resistant organisms such as *D. radiodurans* often exhibit resistance exceeding the applied irradiation dose (up to 15 kGy). Therefore, comparisons with literature values, particularly for *D. radiodurans*, should be interpreted with caution due to differences in irradiation range, source, and experimental design.

A parallel to our results can be drawn to *K. rhizophila* PT10, which showed ~20% survival at 1.0 kGy, a *D*_10_ of ~1.5 kGy, and similar levels of gamma irradiation and desiccation resistance (Guesmi et al., 2021). Despite their contrasting ecological origins in arid plant roots and chronically radioactive springs, both strains exhibit comparable resistance phenotypes, may select for similar stress-associated phenotypes and genomic features within *K. rhizophila*.

Importantly, the pronounced difference between strain 301 and the type strain TA68 supports that pronounced radiation resistance is not uniformly distributed across *K. rhizophila* strains and may reflect local adaptation to extreme environmental conditions. The convergence of resistance phenotypes between strain 301 and PT10, despite their contrasting ecological origins, suggests that chronic low-level radiation exposure in radon-rich environments vs. acute high-dose irradiation used for strain selection may favor the emergence of similar protective phenotypes and associated genomic adaptations. Strain 301 originates from a persistently radiation-exposed environment, where long-term oxidative and DNA-damaging stress likely imposes sustained selective pressure over extended timescales. In contrast, strains such as PT10 or RF are typically isolated following acute irradiation-based selection, representing short-term exposure to high radiation doses. These conditions may therefore reflect different adaptive trajectories, driven by sustained environmental pressure vs. episodic stress. Nevertheless, the observed similarities in genomic features suggest that core protective mechanisms, particularly those related to DNA repair and oxidative stress mitigation, may represent common solutions to radiation-induced damage across these distinct contexts.

### 3.4 Genomic basis of DNA repair

**Figure 4:**
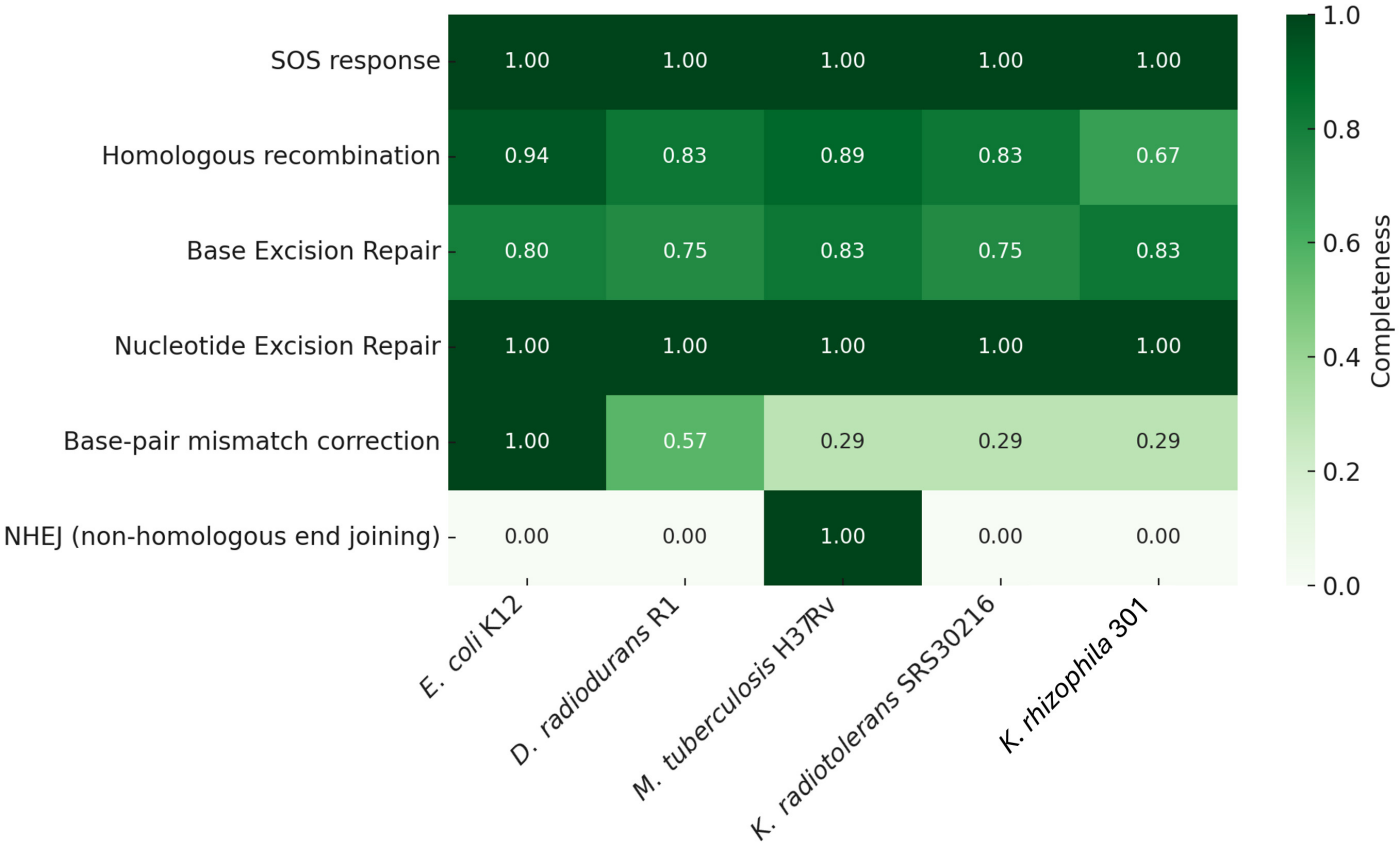
Comparison of gene representation across major DNA repair pathways (SOS, homologous recombination, BER, NER, MMR, NHEJ) in the genomes of selected bacterial strains. Pathway completeness represents the proportion of annotated genes associated with a given repair system present in the genome. A value of 1.0 indicates that all components of the pathway were identified based on genome annotation.

The elevated survival of *K. rhizophila* 301 under gamma irradiation suggests the presence of efficient mechanisms for DNA repair and protection. To examine its genetic capacity, we compared the repertoire of DNA repair genes *K. rhizophila* 301 with those of *E. coli* K12 (Van Houten, 1990; Li, 2008; Ðermić, 2015; Kowalczykowski et al., 1994; Webb et al., 1997; Kuzminov, 1999; Janion, 2008), *D. radiodurans* R1 (Timmins and Moe, 2016; Minton, 1994), *M. tuberculosis* H37Rv (Davis et al., 2002; Della et al., 2004; Ng et al., 2004), and *Kineococcus radiotolerans* SRS30216 (Bagwell et al., 2008; Phillips et al., 2002), species representing distinct phylogenetic groups and levels of radiation resistance. Genes were categorized into major DNA repair and stress-response systems including: HR, BER, NER, MMR, NHEJ, and SOS-associated regulation (Pavlopoulou et al., 2016) (Figure 4). Pathway completeness represents the proportion of annotated genes associated with a given repair system present in the genome; a value of 1.0 indicates that all components of the pathway were identified based on genome annotation and does not imply functional activity.

All compared strains, including *K. rhizophila* 301, encoded the conserved regulators RecA and LexA indicating the genetic potential for an SOS response, although functional activity cannot be inferred from genome annotation alone (Dapa et al., 2017; Janion, 2008; Davis et al., 2002). Taken together, these observations suggest that strain 301 retains the core regulatory architecture associated with bacterial DNA damage responses while lacking specialized *Deinococcus*-type repair modules. Notably, *K. rhizophila* 301 lacks *Deinococcus*-specific genes such as *pprA* and *ddrA–D*, which mediate DNA end stabilization and genome reassembly (Tanaka et al., 2004). This observation suggests that radiation tolerance in strain 301 may rely on reinforcement of conserved repair and stress-response systems rather than on specialized *Deinococcus*-type repair mechanisms.

The homologous recombination machinery comprised *recA, recB, recF, recO, recR*, a putative *recD homolog, ruvC, rarA*, and *radA*, but lacked *recC*, suggesting differences in recombination-related pathways, potentially involving RecFOR-dependent mechanisms (Morimatsu and Kowalczykowski, 2003; Satoh et al., 2012). This configuration parallels that of *K. rhizophila* PT10, while *D. radiodurans* lacks the RecBCD system and relies on alternative recombination pathways. In contrast, *K. radiotolerans* retains both RecBCD and RecFOR systems (Bagwell et al., 2008).

Genes associated with BER (*alkA, mpg, mutY, fpg, mug*) were present, with multiple *fpg* homologs, supporting enhanced repair of oxidative lesions (Tchou et al., 1994). Such redundancy mirrors patterns observed in other radiation-resistant Actinomycetota and supports survival under chronic oxidative stress typical of uranium-rich environments (Pal et al., 2024).

The NER pathway was fully represented (*uvrA, uvrB, uvrC, uvrD*), with three *uvrA* and four *uvrD* paralogs, together with *cho*, suggesting increased redundancy within the excision repair machinery (Perera et al., 2016). By contrast, canonical MMR genes (*mutS, mutL, mutH*) were absent, leaving only *vsr*, a configuration consistent with other actinobacteria (Bagwell et al., 2008; Perera et al., 2016; Guesmi et al., 2021). No *ku* or *ligD* homologs were detected, suggesting the absence of classical NHEJ components. Thus, based on genome annotation, we propose that DNA double-strand break repair in strain 301 involves recombination-based mechanisms, potentially reinforced through redundancy rather than alternative pathways.

### 3.5 Antioxidant defense and protein protection

**Figure 5:**
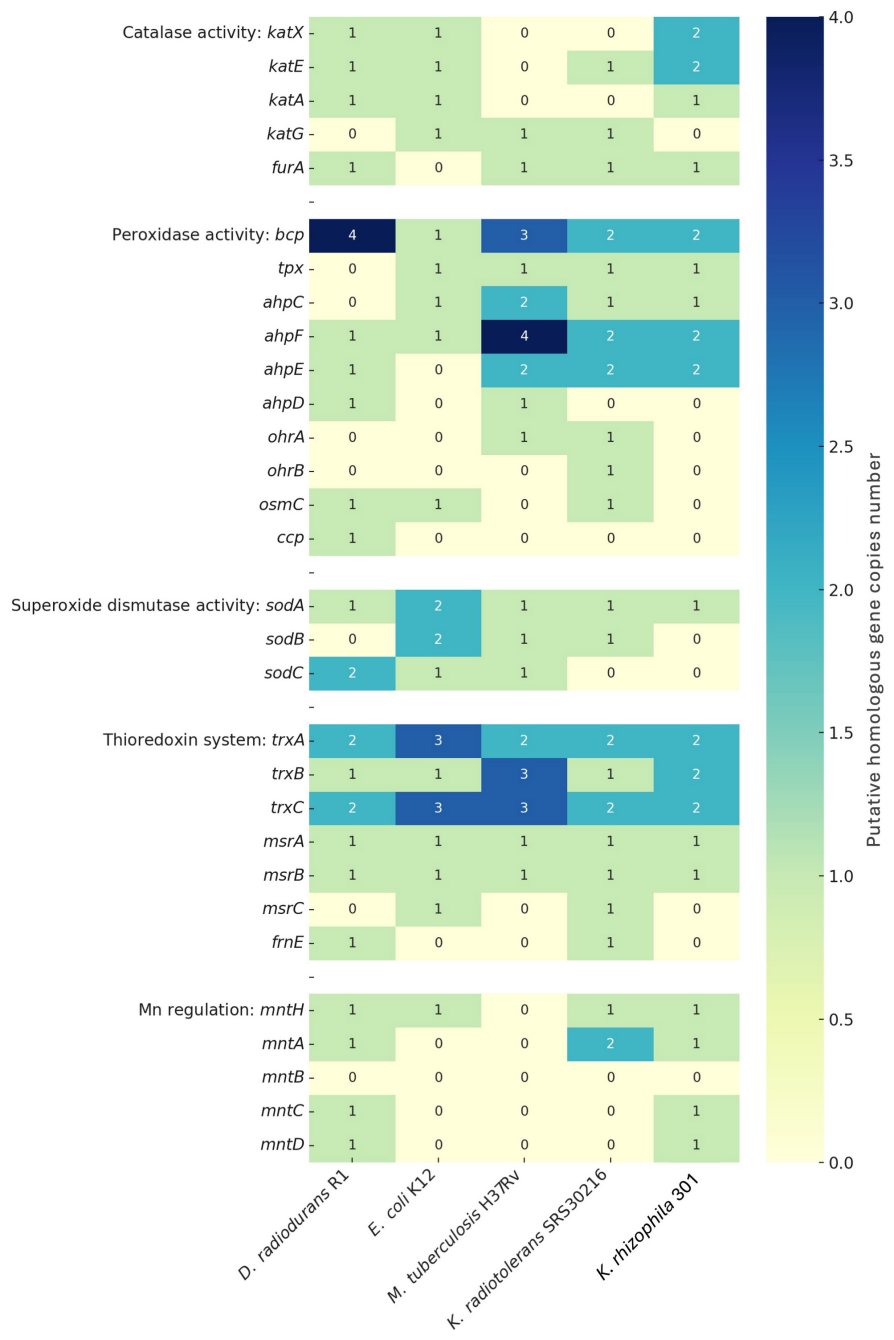
Distribution of genes encoding antioxidant enzymes and Mn^2+^ transporters in selected bacterial strains.

Radiation resistance is known to depend not only on gene content but also on protein-level protection mechanisms, particularly resistance to oxidative damage, as highlighted in previous studies. In this context, ionizing radiation generates reactive oxygen species (ROS), which often inflict lethal damage on proteins and membranes (Daly et al., 2007). *K. rhizophila 301* encodes a broad array of genes putatively associated with enzymatic and non-enzymatic antioxidant systems (Figure 5). These include superoxide dismutases (*sodA, sodB*), catalase–peroxidases (*katG, katA, katE*, each duplicated), and alkyl hydroperoxide reductase (*ahpC*), representing components of a putative multi-layered ROS detoxification system (Nicholls et al., 2001). These differences in gene copy number represent genomic potential and do not directly reflect gene expression levels or protein activity, and therefore cannot be interpreted as evidence of functional activity under radiation stress.

Additional redox-balancing systems include thioredoxins (*trxA, trxB*), glutaredoxin-like proteins, and methionine sulfoxide reductase (*msrA*). Genes involved in manganese uptake and storage were also identified, suggesting a potential capacity for manganese-associated oxidative stress protection, as previously proposed in radiation-resistant bacteria. Such protein-centered protection mechanisms are known to preserve enzymatic function under oxidative stress and indirectly safeguard DNA repair pathways, a strategy well established in *D. radiodurans* although functional equivalence cannot be inferred, and increasingly recognized as contributing to radiation resistance (Fredrickson et al., 2008; Daly, 2009).

### 3.6 Origin and ANIb analysis

**Figure 6:**
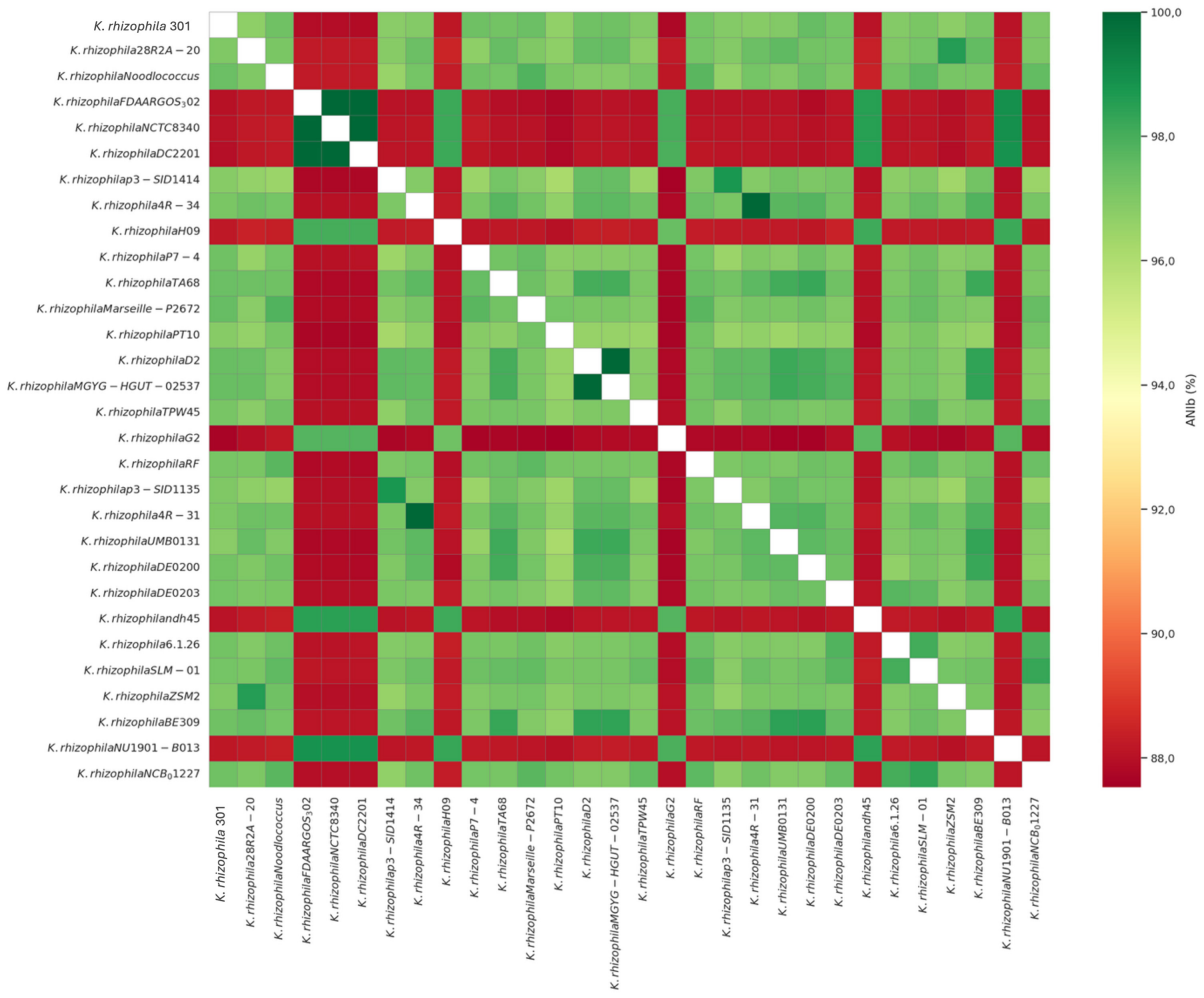
Average Nucleotide Identity based on BLAST (ANIb) similarity between *K. rhizophila* 301 and representative *K. rhizophila* genomes selected from the NCBI database. ANIb values are shown as pairwise genome similarity between strain 301 and selected reference genomes. Higher ANIb values indicate greater genomic similarity.

Pan-genome analysis was performed to assess the genetic diversity of *K. rhizophila* in relation to isolate 301. Based on genome quality and origin, 29 genomes were initially selected. Average nucleotide identity (ANIb) analysis revealed seven genomes with values ≤ 88% (Figure 6), well below species thresholds (Goris et al., 2007; Richter and Rosselló-Móra, 2009). These genomes formed a distinct lineage and were excluded from further analysis to maintain species-level coherence of the dataset for pan-genome reconstruction based on established ANI thresholds.

Conversely, several genomes showed ANIb values approaching 100% in comparison with *K. rhizophila* 301. To avoid redundancy, only a single representative from these highly similar sequences was retained in downstream analyses, selected based on highest genome completeness and assembly quality. Ultimately, genomes with high relatedness to *K. rhizophila* 301 were selected, representing a broad ecological spectrum. The analysis therefore included 21 genomes in total, comprising strain 301 and 20 closely related reference genomes.

The ecological origins of these strains reflect the broad ecological distribution of *K. rhizophila* (Table 3). Isolates have been recovered from human and animal microbiota (e.g., strains SLM-01, D2, UMB0131, p3-SID1135, p3-SID1414, P7-4, and 6.1.26), from soils and geological substrates (e.g., strains RF, 4R-34, and ZSM2), and from aquatic and marine environments including freshwater ecosystems and deep-sea organisms (e.g., strains TPW45, TA68, and 28R2A-20). Colonization of plant-associated niches, particularly the rhizosphere and endophytic habitats (e.g., strains TA68 and PT10), indicates a potential association with plant-related environments or opportunistic lifestyles, although functional interaction cannot be directly inferred from genomic data alone. Other isolates originate from anthropogenic or laboratory settings (e.g., DE0200, DE0203, *Noodlococcus*, Marseille-P2672, and BE309), highlighting the apparent resilience of this species in environments heavily influenced by human activity.

**Table 3:** Average nucleotide identity based on BLAST+ (ANIb) of *K. rhizophila* 301 vs. selected genomes from NCBI database.

Strain	ANIb [%]	Alignment [%]	Origin	Accession (NCBI)
*K. rhizophila* DE0200	97.47	90.16	Environmental sample, Duke University campus	GCF_007677945.1
*K. rhizophila* Marseille-P2672	97.4	89.85	Not specified	GCF_904423785.1
*K. rhizophila* P7-4	97.33	90.95	Gut of fish (*Siganus doliatus*), Pacific Ocean	GCF_000214115.1
*K. rhizophila* D2	97.31	86.83	Human stool (*Homo sapiens*)	GCF_002879775.1
*K. rhizophila* Noodlococcus	97.28	89.61	Laboratory contaminant (University of Birmingham)	GCF_023373505.1
*K. rhizophila* TA68	97.25	86.88	Hungary: Soroksár river, rhizosphere of *Typha angustiflora*	GCF_003667225.1
*K. rhizophila* DE0203	97.21	88.85	Environmental sample, Duke University campus	GCF_007677895.1
*K. rhizophila* RF	97.19	89.86	Soil, Iran	GCF_001190985.1
*K. rhizophila* BE309	97.15	86.23	Not specified	GCA_031457995.1
*K. rhizophila* SLM-01	97.12	87.96	Pathogenic/clinical or host-associated sample	GCF_027889775.1
*K. rhizophila* NCB_01227	97.06	87.80	Not specified	GCF_035990065.1
*K. rhizophila* TPW45	96.94	87.02	Waterfall, Malaysia	GCF_000813865.1
*K. rhizophila* UMB0131	96.94	87.84	Female urinary microbiota	GCA_002861865.1
*K. rhizophila* 6.1.26	96.93	86.89	Skin mucus of zebrafish (*Danio rerio*)	GCF_024655785.1
*K. rhizophila* 4R-34	96.9	84.71	Soil and rock environment	GCF_004563775.1
*K. rhizophila* p3-SID1135	96.89	86.59	Human (*Homo sapiens*), nasal swab	GCF_025144265.1
*K. rhizophila* p3-SID1414	96.81	86.43	Human (*Homo sapiens*), interdigital foot swab	GCF_025151645.1
*K. rhizophila* ZSM2	96.76	87.23	Rock and sandy substrate, Gara Djebilet iron mine (Algeria)	GCF_030403625.1
*K. rhizophila* 28R2A-20	96.72	87.44	Deep-sea sponge	GCA_017723815.1
*K. rhizophila* PT10	96.67	88.00	Endophyte of Saharan plant (*Panicum turgidum*), Tunisia	GCF_900576785.1

Total length of K. rhizophila 301 genome 2,742,214 [bp].

The *K. rhizophila* pan-genome thus provides a framework to assess genetic diversity, functional adaptation, and evolutionary relationships. This ecological versatility spans from soil and water habitats through host-associated niches to extreme or anthropogenic environments. Notably, several isolates have demonstrated pronounced resistance to ionizing radiation and other stressors, including strain PT10, isolated from the roots of the xerophytic plant *Panicum turgidum* in the Tunisian Sahara (Guesmi et al., 2021), and strain RF, obtained from Iranian soil after exposure to 5 kGy of γ-radiation (Mehrabadi et al., 2016). These strains provide valuable genomic and physiological comparators for interpreting the resistance mechanisms of *K. rhizophila* 301, although mechanistic conclusions remain indirect.

Despite this ecological breadth, strain 301 forms a distinct phylogenetic lineage while remaining embedded within a genomically cohesive species framework, indicating adaptive divergence without indication that extensive horizontal gene acquisition represents the primary driver of adaptation This does not exclude the presence of horizontally transferred genes, but suggests adaptive divergence is primarily associated with modification of conserved genomic features, although horizontal gene transfer may still contribute to strain-specific variation.

### 3.7 Pan-genome analysis

Building on the selection of representative genomes based on ANIb values, a pan-genome analysis was performed to explore the genetic diversity and functional repertoire of *K. rhizophila*. A total of 21 high-quality genomes (including *K. rhizophila* 301; see Table 3) were retained for this comparative framework. Clustering of predicted coding sequences was carried out with thresholds of 80% amino acid identity and 80% alignment coverage, ensuring consistent family assignment across datasets. In total, 50,268 predicted genes were grouped into 4,214 gene families, representing the species-level pan-genome of *K. rhizophila*.

**Figure 7:**
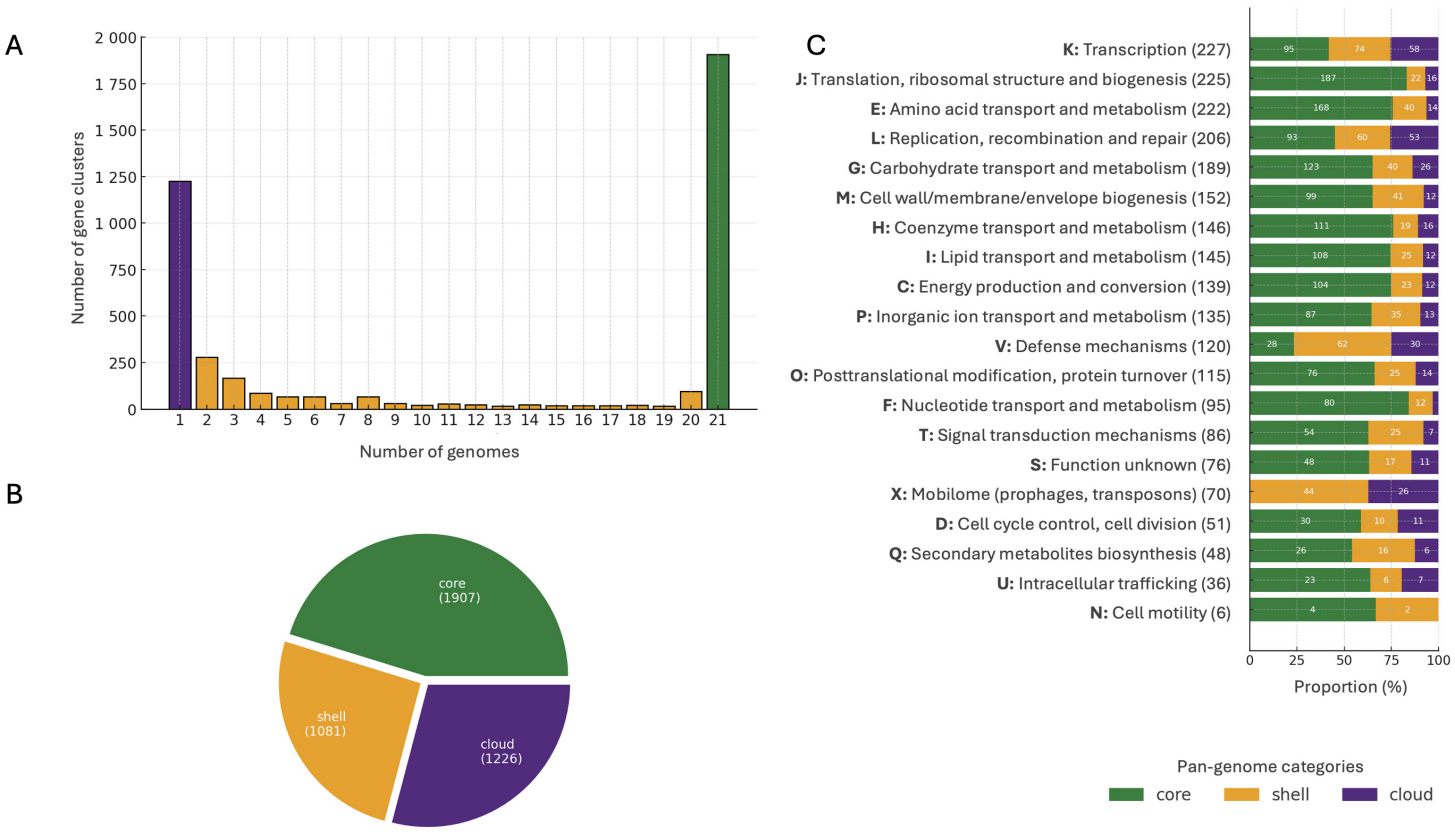
Visualization of the composition and functional diversity of the *K. rhizophila* pan-genome. **(A)** Distribution of gene families according to their occurrence across analyzed genomes. **(B)** Relative proportions of core, shell, and cloud gene fractions, where core genes are present in all genomes, shell genes are present in multiple but not all genomes, and cloud genes are rare or strain-specific. **(C)** Functional categorization of gene families based on COG classification. Core genes are predominantly associated with essential cellular functions, while shell and cloud fractions are enriched in genes related to environmental adaptation and genome plasticity.

Nearly half of all gene families (~45%) were conserved across all genomes, indicating a substantial and stable core component (Figures 7A, B). Gene accumulation modeling yielded parameter estimates of γ = 0.094 and α = 1.088, consistent with a closed pan-genome within the analyzed dataset. The inclusion of additional genomes was predicted to result in only minimal increases in novel gene families, highlighting the possibility of a relatively limited detectable contribution of horizontally acquired gene families within the analyzed dataset, together with the high degree of genomic stability characteristic of this species (Tettelin et al., 2008). The observed closed pan-genome pattern should nevertheless be interpreted within the limits of currently available genome sampling.

Functional annotation of gene families provided further insight into this distribution (Figure 7C). Core genes were predominantly associated with essential cellular processes, including transcription (COG K), translation and ribosome biogenesis (COG J), and DNA replication and repair (COG L). In contrast, shell and cloud fractions were enriched for functions linked to environmental adaptation, such as defense mechanisms (COG V), mobile genetic elements (COG X), transport of ions and metabolites (COGs P, G, H), and secondary metabolism (COG Q). However, these gene families represent strain-specific or low-frequency genomic features, and their direct involvement in radiation resistance cannot be inferred from the current data. Some of these genes may be associated with horizontal gene transfer, as suggested by their functional categories and genomic context; however, this remains putative.

**Figure 8:**
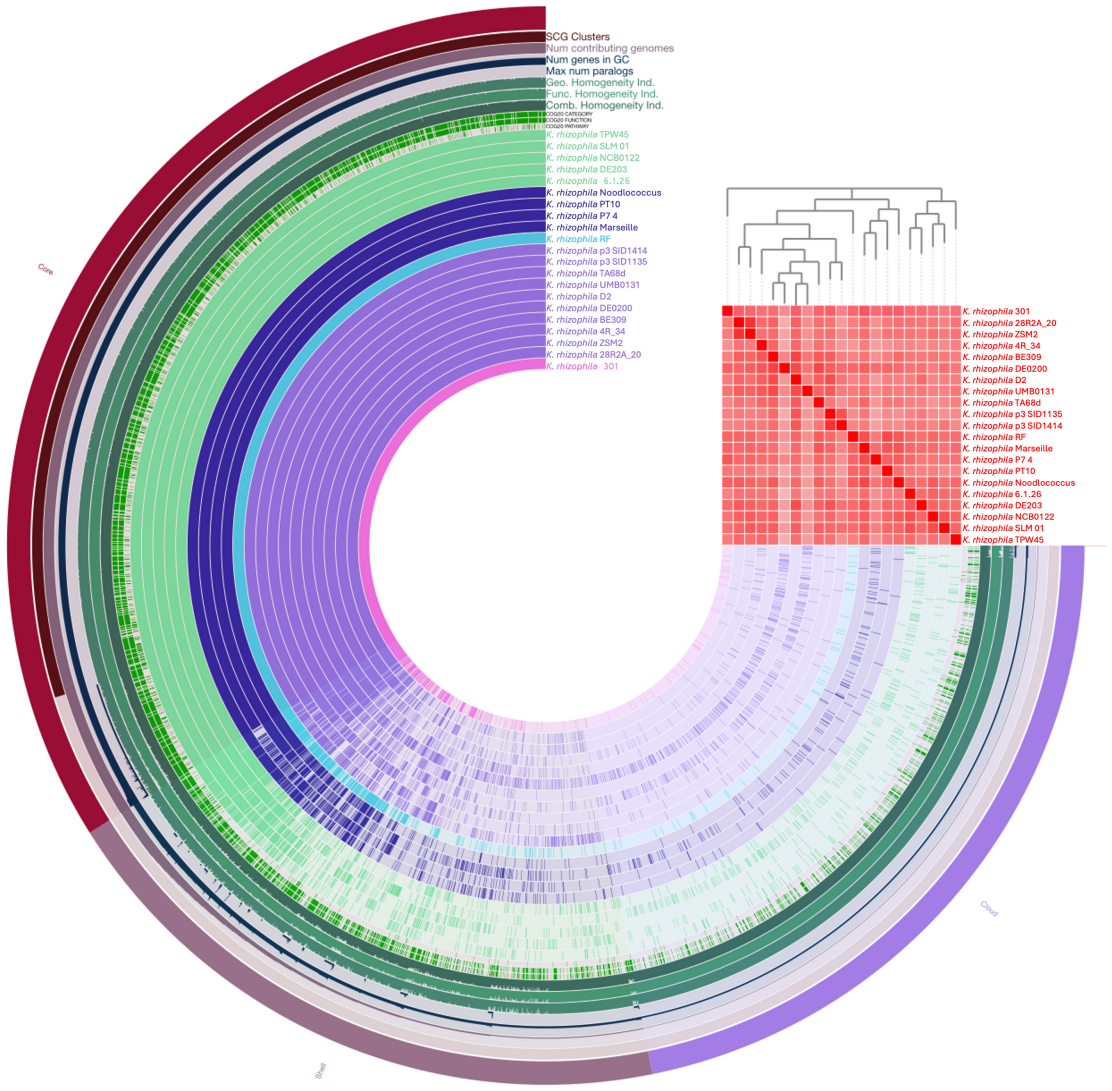
Pan-genome analysis of 21 *K. rhizophila* genomes using Anvi'o. The circular map displays the distribution of core, shell, and cloud gene clusters across the analyzed genomes. Genomes are organized based on clustering using single-copy core genes (SCGs), with colored labels indicating individual genome clusters. The inner layers represent gene presence/absence patterns across genomes. An ANIb heatmap with a hierarchical clustering dendrogram is shown in the top right, illustrating genomic similarity among the analyzed strains.

Phylogenetic analysis based on single-copy core genes (SCG) was further employed to clarify evolutionary relationships within the species. The results showed that *K. rhizophila* 301 diverges from the remaining genomes already at a basal branch of the phylogenetic tree, where it forms a distinct, well-separated monocluster (highlighted in pink in Figure 8). Besides this monocluster, three additional phylogenetically distinct clusters were identified. Cluster A (purple and light blue) comprised eleven genomes, mostly derived from soil, aquatic environments, or human microbiota, including strains 28R2A-20 (deep-sea sponge), ZSM2 (iron mine, Algeria), 4R-34 (soil/rock), BE309 (unspecified), DE0200 (Duke University campus), D2 (human feces), UMB0131 (urinary microbiota), TA68 (rhizosphere), p3-SID1135 and p3-SID1414 (human skin), and RF (Iranian soil). Cluster B (dark blue) included four strains of diverse origin: Marseille-P2672 (unspecified), P7-4 (marine fish gut), PT10 (plant endophyte), and Noodlococcus (laboratory contaminant). The remaining five strains grouped in cluster C (green), comprising isolates 6.1.26 (zebrafish skin mucus), DE0203 (Duke University campus), NCB_01227 (unspecified), SLM-01 (clinical sample), and TPW45 (Malaysian waterfall). The placement of TPW45 outside Cluster A, despite its aquatic origin, suggests that environmental origin alone does not consistently explain clustering patterns in the phylogenetic analysis; however, this observation should be interpreted cautiously, as clustering reflects overall genomic similarity rather than direct ecological or functional equivalence among strains. This study has several limitations. The analysis is based on a limited set of genomes selected for their relatedness to strain 301, which may influence clustering patterns and pan-genome structure. In addition, conclusions are derived primarily from genome annotation and comparative genomics, without functional validation of gene activity. Therefore, the observed associations should be interpreted as indicative of potential adaptive strategies rather than direct evidence of underlying mechanisms.

The genomic analysis of *K. rhizophila* indicates that, although this species occurs in a wide variety of habitats, its adaptability is not clearly associated with an increased proportion of accessory genes or readily identifiable horizontally acquired gene families within the limits of the current dataset or a large accessory genome. Rather, its ecological versatility may be associated with flexible utilization and reinforcement of conserved core genomic functions rather than an expansion of novel gene content, with additional support from selective gene duplications that provide redundancy and increase the dosage of key functions. A partial repertoire of competence-associated genes (e.g., *pilB, pilT, comEA, comEC*, and *dprA*) was also identified in strain 301, although the absence of several key DNA uptake components suggests that a complete competence system is either incomplete or highly divergent (Sharma et al., 2025).

A prominent example is the repertoire of DNA repair genes (Table 4). The core genome of *K. rhizophila* comprises a rich set of 88 conserved genes associated with replication, recombination, and DNA repair. In *K. rhizophila* 301, these functions are reinforced by duplications of *uvrA, ung, yaaA, ssb*, and several helicases, together with additional repair factors in the shell genome such as *phrB* and *recD*. Such redundancy may contribute to maintaining robustness of essential repair processes even under protein-damaging conditions. This pattern is consistent with observations in *D. radiodurans*, where multiple copies of repair genes accelerate genome restoration and safeguard the replication machinery from irreversible mutational damage (Timmins and Moe, 2016).

**Table 4:** Functional repertoire of the *K. rhizophila* pan-genome: core and cloud genes with duplication patterns in *K. rhizophila* 301.

Category	Subgroup	Genes	Function	Gene duplication
Replication/repair	DNA glycosylases	*alkA, ung/udg4, mug, mutY, mpg, nei, nth*	BER—remove damaged/misincorporated bases	*ung/udg4*: 2× in 301
	Endonucleases	*nucS, yraN, rnhA, rnhB*	Cleave DNA at damage sites	–
	Excinucleases	*uvrA, uvrB, uvrC*	NER system	*uvrA*: duplicated in all strains; 2× in 301
	Recombinases	*recA, recG, recN, recR, recO, ruvA, ruvB, ruvC*	Homologous recombination, DSB repair	–
	Topoisomerases	*gyrA, gyrB*	DNA unwinding	*gyrA*: 2× in 301
	Polymerases	*dnaQ, dnaE, dnaX, dnaN, polA*	Replication synthesis/proofreading	–
	Helicases	*uvrD, dinG, recQ, srmB, herA, lhr, yprA*	DNA unwinding	*helD*: 2× in 301
	Exonuclease complex	*sbcC, sbcD*	Remove damaged/aberrant DNA	–
	Ligase	*lig*	Seal DNA breaks	–
	301-specific duplications	*tag, yaaA, ssb, mod, dprA/smf, helD, udg4*	DNA repair/protection functions	All 2× only in 301
Defense	ABC transporters (permeases)	*yadH, salY, emrE, lolC*	Export antimicrobial compounds/peptides	*salY*: 4× in 301; *lolC*: 4× in 301; *yadH*: 2× in 301
	ABC transporters (ATPases)	*mdlB, ccmA*	Provide energy for export	–
	Efflux pumps	*dinF, norM, emrE*	Multidrug transporters	*emrE*: 2× in 301
	NUDIX hydrolase	*mutT*	Cleave oxidized nucleotides (8-oxo-dGTP)	–
	Restriction-modification	*hsdM, hsdS*	Type I R-M system	*hsdM*: 2× in 301
	Deglycase	*yajL*	Repair glyoxal-modified DNA/proteins	*yajL*: 2× in 301
	Nitro-reductase	*nimA*	Detoxify nitroimidazoles	–
	Enamine deaminase	*ridA*	Detoxify reactive intermediates	–
Secondary metabolism	Aromatic degradation	*catE/gloA, ycgM/mhpD, paaB, ydbO, paaI, acuC*	Catechol and phenylacetate pathway enzymes (dioxygenases, hydratases, thioesterases, deacetylases)	–
	Biotin synthesis	*cypX*	Cofactor biosynthesis	2 paralogs
	Stress detox	*dinB, ara1, maf*	Detox of electrophiles	–
	Surfactant synthesis	*grsT*	Biosurfactant	–
	Carotenoid synthesis	phytoene dehydrogenase family protein	Pigment precursors	–
Ion transport	Fe transport (siderophore)	*fepC/afuC, fsr, fdx*	Import and reduce Fe^3+^ complexes	*fepC*: 2× in 301; *viuB*: 2× in 301
	Mn transport	*mntH*	NRAMP transporter	–
	Mn/Zn transport	*znuC*	ABC transporter	–
	Fe^2+^/Mn^2+^ transport	*feoB-like*	Membrane symporter	–
	Siderophore synthesis	*lucA/iucA*	Siderophore ligands	–
	Metal detox	*czcO, czcD, copZ, arsB, terC*	Efflux systems	*czcD*: 3×; *czcO*: 2×; *copZ*: 3×; *arsB*: 2×; *terC*: 2× in 301
Cloud genome *K. rhizophila* 301	Defense/repair	*hsdR, hsdM, ytxK*	Restriction-modification	–
	Carbohydrate metabolism	*nagA, nagB, ptsG*	N-acetylglucosamine utilization	–
	Regulators/others	*gntR, rodZ*	Transcription factor, morphogenesis	–
	Unannotated	16 genes	Unknown	–

A similar principle was observed in defense and detoxification genes. Core functions include multidrug transporters, ABC permeases, and restriction–modification systems. *K. rhizophila* 301 again shows broad duplication, with multiple paralogs of *salY, lolC, emrE*, and *hsdM*, pointing to enhanced efflux capacity and protection against phage DNA. Rather than introducing novel resistance modules, the bacterium appears to rely on amplification of existing defenses, potentially increasing their quantitative impact.

Homologs of serine/threonine protein kinases were identified in the shell genome of *K. rhizophila*, including strains 301 and PT10, suggesting their presence as accessory regulatory features. Although relatively rare in bacteria, these kinases have been implicated in stress sensing and regulation of cellular repair pathways (Rajpurohit et al., 2022; Sharma et al., 2022a, 2020; Soni et al., 2026). Their potential contribution to stress adaptation in strain 301, however, remains to be experimentally validated.

The most striking adaptation is found in metal ion homeostasis. The core genome of *K. rhizophila* encodes a wide spectrum of systems for metal uptake and efflux. These include ABC transporters for siderophores and Fe^3+^ (*fepC, afuC*), NRAMP transporters MntH for Mn^2+^/Fe^2^^+^, ABC transporter subunit ZnuC for Zn^2+^/Mn^2+^, and efflux pumps CzcO and CzcD for Co^2+^/Zn^2+^/Cd^2+^ (Hohle and O'brian, 2009; Patzer and Hantke, 1998; Nies, 1992). *K. rhizophila* 301 shows an expanded repertoire of these systems, with three paralogs of *copZ* and three paralogs of *czcD*. In its shell genome, additional Fe^2+^ uptake genes *feoA/feoB*, an extra *mntH*, up to four *cadD* paralogs (cadmium resistance), and supplementary *copZ* paralogs were identified. Such functional redundancy may enable rapid responses to fluctuations in metal availability or to toxic concentrations. A similar profile is found in strain PT10, which shares with *K. rhizophila* 301 several shell genes for metal transport (*mntH, copZ, feoAB, zupT, zntA, cadD, and arsB*) and also encodes siderophore biosynthesis genes and TRASH/YHS domain metallochaperones (Guesmi et al., 2021). These proteins allow safe intracellular distribution of metals without triggering harmful redox reactions, a feature critical for survival under high radiation and iron limitation.

The strategy is even more pronounced in the extremely radioresistant *K. radiotolerans*, whose genome encodes multiple paralogs of *mntH* and *feoAB*, extensive efflux systems (*czcD, copA/copZ, arsB, and cadA*), and complete siderophore biosynthetic pathways (Bagwell et al., 2008). This strain combines a high Mn/Fe ratio, typical for *D. radiodurans*, with active detoxification of heavy metals, thereby minimizing oxidative damage to proteins and DNA during irradiation. High Mn^2+^ accumulation is particularly important: Mn^2+^ complexes protect proteins against oxidation, preserving the function of DNA repair enzymes after irradiation (Daly, 2009). At the same time, active efflux of metals with pro-oxidant potential (Fe^2+^, Cu^2+^, Co^2+^, Cd^2+^) is associated with cross-protection against oxidative stress.

The present comparative analysis suggests that ecological breadth in K. rhizophila may depend more strongly on the flexible utilization and reinforcement of conserved genomic functions than on the acquisition of novel genes through horizontal gene transfer. *K. rhizophila* 301 exemplifies this strategy: gene duplications across DNA repair, defense, and ion homeostasis pathways may reinforce essential processes and buffer against environmental stress.

This study suggests that the observed radiation resistance of *K. rhizophila* 301 correlates with increased copy numbers of genes involved in DNA repair, antioxidant defense, and metal ion homeostasis. While this relationship is supported by consistent phenotypic and genomic patterns, it also highlights an important direction for future research. Functional validation of this association, for example through targeted gene disruption, controlled overexpression, or transcriptomic and proteomic analyses under irradiation conditions, would enable direct assessment of the contribution of gene dosage to the resistant phenotype. Such approaches will allow confirmation whether the observed genomic reinforcement translates into increased activity of key protective systems and will provide a mechanistic framework linking genome architecture to stress tolerance. These results provide a basis for future comparative analyses across multiple *K. rhizophila* strains to assess whether increased gene copy number consistently associates with radiation resistance.

**Figure 9:**
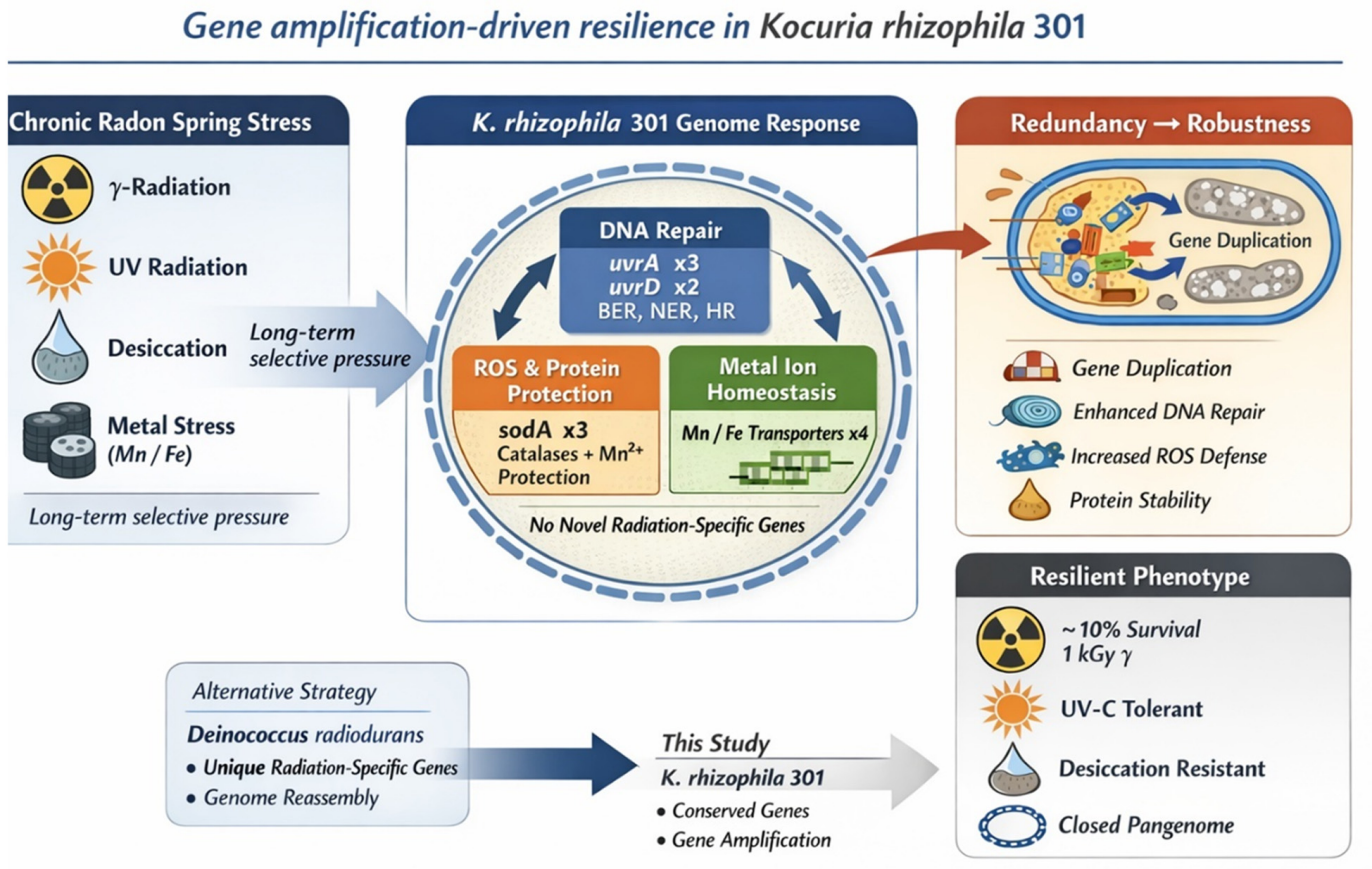
Conceptual model of radiation tolerance in *K. rhizophila* 301 based on the proposed mechanisms contributing to radiation tolerance in strain 301 based on genomic and phenotypic analyses. Key processes include enhanced DNA repair capacity, antioxidant defense systems, and metal ion homeostasis, supported by increased gene copy numbers of conserved stress-response genes.

Based on the combined phenotypic and genomic data we propose a conceptual model in which chronic exposure to ionizing radiation may select for amplification or preferential retention of conserved stress-response pathways rather than the acquisition of radiation-specific genes (Figure 9). In this model, *K. rhizophila* 301 the increased gene dosage across DNA repair, antioxidant defense, and metal ion homeostasis enhances cellular robustness, which may contribute to functional robustness of core cellular processes under environmental stress conditions.

Several limitations of the present study should be acknowledged. The proposed contribution of duplicated repair, antioxidant, and metal homeostasis genes to radiation resistance is based primarily on comparative genomic inference and correlation with the observed phenotype, and therefore does not demonstrate causal relationships between gene dosage and functional stress resistance. Functional validation of individual genes, transcriptional responses during irradiation, intracellular Mn/Fe ratios, and protein oxidation dynamics were beyond the scope of this study. Similarly, the presence of homologous genes does not necessarily imply conservation of regulatory mechanisms or pathway activity. Future transcriptomic, proteomic, and mutational analyses will therefore be necessary to determine the precise contribution of these systems to stress tolerance in *K. rhizophila* 301.

## 4 Conclusion

In this study, we propose that radiation resistance in *K. rhizophila* is primarily associated with the amplification of conserved genetic pathways rather than the acquisition of novel, radiation-specific genes. Strain 301, isolated from chronically radioactive radon springs, exhibits pronounced tolerance to ionizing radiation and desiccation, accompanied by increased copy numbers of genes involved in DNA repair, oxidative stress defense, and metal ion homeostasis.

The absence of clearly identifiable lineage-specific resistance determinants within the analyzed dataset, together with a closed species pan-genome under current sampling, suggests that adaptive potential in *K. rhizophila* may be shaped by quantitative modulation of its core genome. This pattern differs from well-characterized paradigms derived from *Deinococcus* species and may represent an alternative evolutionary strategy in which robustness emerges from redundancy and gene dosage effects rather than *de novo* genetic innovation.

By integrating ecological context with phenotypic and comparative genomic analyses, our findings highlight the potential role of long-term environmental pressure in shaping microbial stress tolerance and position *K. rhizophila* 301 as a useful model for studying convergent stress-response strategies, genome stability, and survival in chronically radioactive habitats. This work also provides a genomic framework for identifying quantitative determinants of stress resilience in Actinomycetota from extreme and contaminated environments. It also establishes a basis for future studies aimed at experimentally testing the role of gene dosage in radiation resistance and linking genomic architecture to functional stress response mechanisms.

## Data Availability

The raw data generated in this study can be found in the NCBI (https://www.ncbi.nlm.nih.gov), BioProject accession PRJNA1402305.
