# Supplementary material for: Genomic signatures of radiation stress adaptations in Kocuria rhizophila: insights from strain 301 of the Jáchymov radon springs
Source: Front Microbiol. 2026 Jul 8;17:1814458. doi: 10.3389/fmicb.2026.1814458 (PMC13388538; doi:10.3389/fmicb.2026.1814458)
Supplement: Supplementary file 1 [file Table_1.docx]

Supplements

Table 1 Biochemical profiling of *K. rhizophila* 301 and *K. rhizophila* TA68 by API ZYM ((bioMérieux, Marcy-l'Étoile, France).

| **API ZYM substrate** | ***K. rhizophila* 301** | ***K. rhizophila* TA68** |
| --- | --- | --- |
| G1 p-Hydroxy- Phenylacetic Acid | Positive | - |
| H1 Tween 40 | - | - |
| A2 Dextrin | - | - |
| B2 a-D-Lactose | - | - |
| C2 D-Mannose | - | - |
| D2 D-Mannitol | - | - |
| E2 Glycyl-L-Proline | Positive | Positive |
| F2 D-Galacturonic Acid | - | - |
| G2 Methyl Pyruvate | - | - |
| H2 g-Amino-Butryric Acid | Positive | - |
| A3 D-Maltose | Positive | - |
| B3 D-Melibiose | - | - |
| C3 D-Fructose | - | - |
| D3 D-Arabitol | - | - |
| E3 L-Alanine | - | - |
| F3 L-Galactonic Acid Lactone | - | - |
| G3 D-Lactic Acid Methyl Ester | - | - |
| H3 a-Hydroxy- Butyric Acid | - | - |
| A4 D-Trehalose | Positive | - |
| B4 b-Methyl-D- Glucoside | - | - |
| C4 D-Galactose | - | - |
| D4 myo-Inositol | - | - |
| E4 L-Arginine | Positive | - |
| F4 D-Gluconic Acid | Positive | - |
| G4 L-Lactic Acid | Positive | Positive |
| H4 β-Hydroxy-D,L- Butyric Acid | Positive | Positive |
| A5 D-Cellobiose | - | - |
| B5 D-Salicin | - | - |
| C5 3-Methyl Glucose | - | - |
| D5 Glycerol | Positive | - |
| E5 L-Aspartic Acid | - | - |
| F5 D-Glucuronic Acid | - | - |
| G5 Citric Acid | - | - |
| H5 a-Keto-Butyric Acid | - | - |
| A6 Gentiobiose | - | - |
| B6 N-Acetyl-D- Glucosamine | Positive | - |
| C6 D-Fucose | - | - |
| D6 D-Glucose- 6-PO4 | - | - |
| E6 L-Glutamic Acid | - | Positive |
| F6 Glucuronamide | - | - |
| G6 a-Keto-Glutaric Acid | - | - |
| H6 Acetoacetic Acid | - | - |
| A7 Sucrose | - | - |
| B7 N-Acetyl-b-D- Mannosamine | - | - |
| C7 L-Fucose | - | - |
| D7 D-Fructose- 6-PO4 | - | - |
| E7 L-Histidine | Positive | - |
| F7 Mucic Acid | - | - |
| G7 D-Malic Acid | - | - |
| H7 Propionic Acid | - | - |
| A8 D-Turanose | - | Positive |
| B8 N-Acetyl-D- Galactosamine | - | - |
| C8 L-Rhamnose | - | - |
| D8 D-Aspartic Acid | - | - |
| E8 L-Pyroglutamic Acid | Positive | - |
| F8 Quinic Acid | - | - |
| G8 L-Malic Acid | Positive | - |
| H8 Acetic Acid | - | - |
| A9 Stachyose | - | - |
| B9 N-Acetyl Neuraminic Acid | - | - |
| C9 Inosine | Positive | - |
| D9 D-Serine | - | - |
| E9 L-Serine | Positive | - |
| F9 D-Saccharic Acid | - | - |
| G9 Bromo-Succinic Acid | - | - |
| H9 Formic Acid | - | - |
| B10 1% NaCl | Positive | Positive |
| C10 1% Sodium Lactate | Positive | Positive |
| D10 Troleandomycin | Positive | - |
| E10 Lincomycin | - | - |
| F10 Vancomycin | - | - |
| G10 Nalidixic Acid | Positive | - |
| H10 Aztreonam | - | - |
| A11 pH 6 | Positive | Positive |
| B11 4% NaCl | Positive | Positive |
| C11 Fusidic Acid | - | - |
| D11 Rifamycin SV | - | - |
| E11 Guanidine HCl | Positive | - |
| F11 Tetrazolium Violet | - | - |
| G11 Lithium Chloride | Positive | Positive |
| H11 Sodium Butyrate | Positive | Positive |
| A12 pH 5 | - | Positive |
| B12 8% NaCl | - | Positive |
| C12 D-Serine | Positive | - |
| D12 Minocycline | - | - |
| E12 Niaproof 4 | - | - |
| F12 Tetrazolium Blue | - | - |
| G12 Potassium Tellurite | Positive | Positive |
| H12 Sodium Bromate | Positive | Positive |
